# Investigation of household private car ownership considering interdependent consumer preference

**DOI:** 10.1371/journal.pone.0219212

**Published:** 2019-07-10

**Authors:** Na Wu, Chunyan Tang

**Affiliations:** 1 School of Highway, Chang’an University, Xi’an city, ShaanXi Province, China; 2 School of Transportation Engineering, Dalian Maritime University, Dalian city, Liaoning Province, China; Huazhong University of Science and Technology, CHINA

## Abstract

People are connected by various social networks, resulting in the interdependence of consumer choice. Therefore, it is very important and realistic to assume choice interdependence in private car ownership modeling. In this paper, we investigate the interdependence of private car ownership choice using a spatial autoregressive binary probit model estimated by the Bayesian Markov chain Monte Carlo (MCMC) method. Constructing the autoregressive matrix demographically shows that the private car ownership choice of a household is dependent on other household choices. Compared with the pure binary probit model estimated by the MCMC method, the spatial autoregressive model achieves a significant improvement both in loglikelihood value and log marginal density value, which are calculated using the importance sampling method of Newton and Raftery, from approximately -202 to approximately -63 and from -208 to -145, respectively. Moreover, the results indicated by the spatial autoregressive probit model suggest that the number of children, the ownership of an apartment or the availability of a parking lot are positively and significantly associated with the private car ownership level. To test the out-of-sample performance of the model, we estimate the model using 600 data points and test it using another 148 data points. The results indicate that the predictive power is greatly improved. Finally, we analyze the augmented parameter and discover that it is associated with the parking variable in addition to the license variable.

## Introduction

Research on household private car ownership has received considerable attention in the fields of economics, marketing and transportation. Three main reasons contribute to this. First, household private car ownership is critical in travel demand analysis. It is widely recognized that private car ownership plays a vital and ubiquitous role in the travel behavior of individuals and households both in long-term decisions, such as residential location, and short-term decisions, such as activity patterns [1] and travel mode choice. Second, studies about private car ownership type choice and usage behavior can provide insights to help vehicle-related organizations or government agencies to make policies. For instance, car manufacturers can use these models to determine their strategies for different demographic subgroups in terms of vehicle production and vehicle type design. Moreover, research also provides a means to analyze the consumer evaluation of vehicle attributes that are not yet on the market. For governments, the models are helpful in investment decision making. For example, in the construction of the metro system, future private car ownership and the capacity of the transportation system of a city are key elements for the State Council of China to evaluate the investment. Third, vehicle type holdings and usage play a significant role in reducing greenhouse emissions and energy consumption. It is well known that transportation is a dominant component of the world’s CO^2^ emissions, and the emissions from private cars are almost half of the total transportation emissions [2]. Out of concern for energy and environmental sustainability, planners and policymakers try to find effective ways to address the impact of vehicular travel and thus deal with environmental problems [3–5].

Models to predict changes in the level of private car ownership have been under development since the 1930s [6]. The earlier research approaches about private car ownership are at the aggregate level [7], investigating ownership at a zonal, regional or national level. The aggregate modeling approach, however, fails to capture the underlying behavioral mechanisms that actually guide the household decision-making process, although it is considered cost-efficient in terms of computation and data requirements. On the other hand, disaggregate models can alleviate these difficulties by investigating the household or individual propensity to own vehicles, thereby providing more precise and detailed suggestions for policy makers [8]. Therefore, in recent years, scholars have examined private car ownership at a disaggregate level (household level). We restrict our focus to these household-level studies.

The methodological approaches used to model private car ownership at the discrete level can be divided into four subgroups: count models, standard discrete choice models, advanced discrete choice models, and other approaches, which refer mainly to the machine learning approach [8]. Since many alternatives have zero choosing probability in the population, count models, such as the Poisson regression and negative binomial regression models, are quite restrictive in real applications. Moreover, because the machine learning algorithm contains many nodes and layers, it is rather limited for application in policy and sensitivity analysis. Thus, we summarize two main aspects of the private car ownership modeling approaches: standard discrete choice models and advanced discrete choice models.

Within the class of standard discrete choice models, the ordered-response and the unordered-response decision mechanisms have commonly been used for private car ownership modeling [9,10]. The ordered response mechanism treats latent utility as a continuous unidirectional variable, while the unordered response mechanism is derived from the random utility maximization (RUM) principle [11], which assumes that households associate a utility value with each private car ownership level and choose the private car ownership level with the highest utility. The ordered response models include ordered logit [12,13] and ordered probit [14,15]. The unordered response models contain a multinomial logit model [16,17], a multinomial probit model, and a nested model. Later, researchers incorporated heterogeneity in the population into the standard discrete choice models, obtaining the random parameters model [18] and the latent class model [19].

Unlimited dependency on private cars results in serious problems, such as traffic congestion and energy consumption. Jointly studying household travel behavior and the related decisions could provide useful insights into reducing car dependency and thus has attracted many researchers [20–23]. To address the endogenous issue in integrated modeling, various advanced discrete choice models have been developed and employed, such as the structural equation model [24–26], multiple discrete continuous extreme value model (MDCEV) [27–29], joint mixed multinomial logit-ordered response structure [30], copula-based model [31–34], and Bayesian model [35–38]. Moreover, to capture the influence of latent variables such as environmental concern, Daziano and Bolduc [37] employed an integrated choice model with a latent variable (ICLV) model [39] to study green vehicle adoption.

Most recently, some researchers found that neighboring households may exhibit preferences that are spatially or demographically similar. This means that a household’s preference can be influenced by others’ preferences, which may be driven by social concerns, peer pressure, or endorsements from respected people. It is discovered that vehicle ownership was spatially dependent [40,41], and social interactions can influence plug-in hybrid electric vehicles (PHEVs) assessment and adoption [42]. Song and Wang [43] found that households with high levels of car ownership presented locally spatial clusters. Ding et al [44] examined the impacts of the built environment on commuter's driving behavior at spatial zone level. Moreover, the structure of a country's car fleet is not only dependent on domestic policies, but is also affected by the policies of neighboring countries using a series of spatial regression models [45].

This paper contributes to the private car ownership literature by using the Bayesian-based spatial autoregressive binary probit model [46] to study the choice interdependence of owning a vehicle or not owning a vehicle across households. The model can be constructed to capture the endogenous nature of preference as well. Since the augmented parameter in the autoregressive process makes posterior distribution draws easier, the Markov chain Monte Carlo (MCMC) Gibbs sampler is employed to estimate the model.

The remainder of the paper is constructed as follows. Section 2 presents the modeling methodology, including model structure and estimation procedures. Section 3 describes the data followed by the results analysis in section 4. Finally, section 5 offers some concluding comments.

## Modeling methodology

### Model structure

In the standard binary probit model, individual *n* (*n = 1*, …,*N*) faces two alternatives (*y*_*n*_ = 1 or 0) and needs to make a selection. The final choice is driven by the difference in latent utilities, *U*_*nk*_(*k* = 1,2 for the binary probit). Therefore, the probability can be written as:

$$\mathrm{Pr}(y_{n}=1)=\mathrm{Pr}(U_{n1}>U_{n2})=\mathrm{Pr}(z_{n}>0),z_{n}=x_{n}^{'}\beta +\varepsilon_{n}$$ (1)

Where *z*_*n*_ is the difference of latent utility between the first alternative *y*_*n*_ = 1 and the second alternative *y*_*n*_ = 0. If *z*_*n*_>0, the first alternative is chosen. *ε*_*n*_~*N*(0,1) for identification. *x*_*n*_ is a vector of variables describing characteristics of the household *n*, and *β* is the vector of coefficients associated with *x*_*n*_. Under this scenario, *ε*_*n*_ is assumed to be distributed independently in the population. However, what if it is not? It is well known that people are social creatures, and people around the world make efforts to establish their own social networks driven by various motivations. These well-established relationships may result in the dependence of *ε*_*n*_ in the population. This study aims to investigate whether this dependence exists in the household behavior of private car ownership.

If the interdependence among households is taken into account, we need to construct the error structure for the complete observations rather than only for all the alternatives of each observation, which is the common practice for the standard probit model. Since only two alternatives exist in our case, *ε*_*n*_ reduces to one element that is specified in Eq 1. Thus, the error structure for all observations is:

$$\varepsilon =\left[\begin{array}{llll} 1 & \varepsilon_{12} & \ldots & \varepsilon_{1N}\\ \varepsilon_{21} & 1 & \ldots & \varepsilon_{2N}\\ \vdots & \vdots & \ddots & \vdots \\ \varepsilon_{N1} & \varepsilon_{N2} & \ldots & 1 \end{array}\right]$$ (2)

Due to the presence of off-diagonal elements, the expectation of latent preference of a household is associated with another household [46]:

$$E[z_{2}|z_{1}]=X_{2}^{'}\beta +\Sigma_{21}\Sigma_{11}^{-1}(z_{1}-X_{1}\beta )$$ (3)

Where Σ_11_ is the covariance matrix of household 1, and Σ_12_ are the covariance matrices between households 1 and 2. We adopt the approach of Yang and Alleby [46] to model the correlation structure. It is restated as follows:

$$z_{n}=x_{n}^{'}\beta +\varepsilon +\theta ,$$ (4)

θ=ρWθ+u, (5)

ε∼N(0,I), (6)

$$u∼N(0,\sigma^{2}I).$$ (7)

Where *ε* reflects the unobserved error, which is assumed to be distributed independently among households, *θ* is a vector of autoregressive parameters, and matrix *ρW* captures the interdependence of preferences across households. Nonzero entries appearing in the nondiagonal of *W* = {*w*_*ij*_} indicate different relationships across households. According to different definitions of a relation, the weight matrix *W* can be defined in multiple ways. Sometimes, the endogeneity of *W* should be considered as well [47]. In this study, we investigate only the interdependence among demographic neighbors. We count the number of driver licenses in a household as an indicator of the relation. We believe there are some commonalities in terms of the desire for a vehicle if this indicator is identical. This dependence may present some similarities with respect to private car ownership across households. The weight matrix is specified as follows: *w*_*ij*_ = 1 if household *i* and household *j* have the same number of driver licenses; otherwise, *w*_*ij*_ = 0. Here, we adopt weighted *W*, in which the diagonal elements are zero and each row sums to one. The coefficient *ρ* measures the endogenous association among households. If *ρ* is positive, then a positive dependence across households exists; otherwise, a negative dependence exists.

Under this model specification, the augmented error model results in a latent preference with nonzero covariance, as shown by Eq (8):

$$z∼N(X\beta ,I+\sigma^{2}{\left(I-\rho W\right)}^{-1}(I-\rho W^{'}{)}^{-1})$$ (8)

The specification of augmented parameter *θ* in this way makes posterior distribution draws of parameters in the model straightforward. Therefore, we employ a Bayesian MCMC Gibbs sampler to estimate the model.

### Model estimation

Bayesian estimation has two main advantages over classical procedures [48]. First, it does not need to maximize any function. Therefore, it relies on the trap caused by initial values or simulation errors, especially for the probit model. Second, desirable estimation properties, such as consistency and efficiency, can be obtained under more relaxed conditions than in classical procedures. In the Bayesian framework, after obtaining the posterior distribution of parameters, we use the MCMC method to draw samples from it. After thousands of iterations, the sample drfaws converges to the true value. The mean and standard deviation of these draws constitute the estimate and standard error. We specify the standard conjugate priors of parameters in the spatial autoregressive binary probit model as follows:

$$\beta ∼N(\bar{\beta },A^{-1}),\bar{\beta }=0,A=0.01I,$$ (9)

$$\rho ∼Unif[\frac{1}{\lambda_{\min}},\frac{1}{\lambda_{\max}}],$$ (10)

$$\sigma^{2}∼\frac{\nu_{0}s_{0}^{2}}{\chi_{\nu_{0}}^{2}},\nu_{0}=5,s_{0}^{2}=0.1.$$ (11)

Since we need to guarantee that *I*−*ρW* is invertible, which appears in the posterior distribution, *ρ* is restricted to lie in the interval [$\frac{1}{\lambda_{\min}},\frac{1}{\lambda_{\max}}$] (here, *λ*_min_<0,*λ*_max_>0), where *λ* denotes the eigenvalue of matrix *I*−*ρW*. Then, a Gibbs sampler is constructed to draw from the conditional posterior (details are listed in appendix):

$$\begin{array}{l} z|\beta ,\theta ,y\\ \beta |z,\theta \\ \theta |\rho ,\sigma^{2},z,\beta \\ \sigma^{2}|\rho ,\theta \\ \rho |\sigma^{2},\beta \end{array}$$

### Data

Dalian city, which is in the Liaoning province of China, is located in the northeastern part of China and is surrounded by the Bohai Sea on three sides. In 2015, it had a population of approximately 5.93 million, and it contained approximately 2.13 million households. The number of drivers increased from 1.79 million in 2014 to 1.97 million in 2015. Moreover, the private car ownership of Dalian in 2015 was over 956 thousand; compared with 847 thousand in 2014, it increased by 12.8%. These statistics show that Dalian is still in the stage of vehicle motorization. In this context, it will be interesting and meaningful to study the influence of social relationships on household private car ownership behavior. Subsequently, we may quantify education’s importance in reducing vehicle reliance and energy consumption by promoting environmental awareness (statistics in this paragraph come from the Dalian Statistical Yearbook 2016).

We collected revealed preference (RP) data by questionnaires (see S1 and S2 Questionnaires) in July 2015 at 12 different sites in Dalian. After excluding incomplete data, we obtained a total of 748 observations. In the questionnaire, household demographics and some details about household private car ownership and usage were recorded. Participants provided written consent for their questionnaire responses to be used for the purposes of scientific research. This study only considered private car ownership modeling. Table 1 lists the statistics of the sample.

**Table 1:** Statistics of the sample.

Variables	Count	Ratio	Variables	Count	Ratio
Ownership			Monthly expense		
0	313	41.8%	Within 5,000 RMB	332	44.4%
1	435	58.2%	5,000–10,000 RMB	324	43.3%
Children			10,000–20,000 RMB	76	10.2%
0	382	51.1%	Above 20,000 RMB	16	2.1%
1	325	43.4%	Number of licenses		
2 or more	41	5.5%	0	86	11.5%
Apartment			1	285	38.1%
0	208	27.8%	2	307	41.0%
1	540	72.2%	3 or more	70	9.4%
Education			Parking		
High school	101	13.5%	0	442	59.1%
College	131	17.5%	1	306	40.9%
Undergraduate	373	49.9%			
Graduate	143	19.1%			

## Results

**Fig 1:**
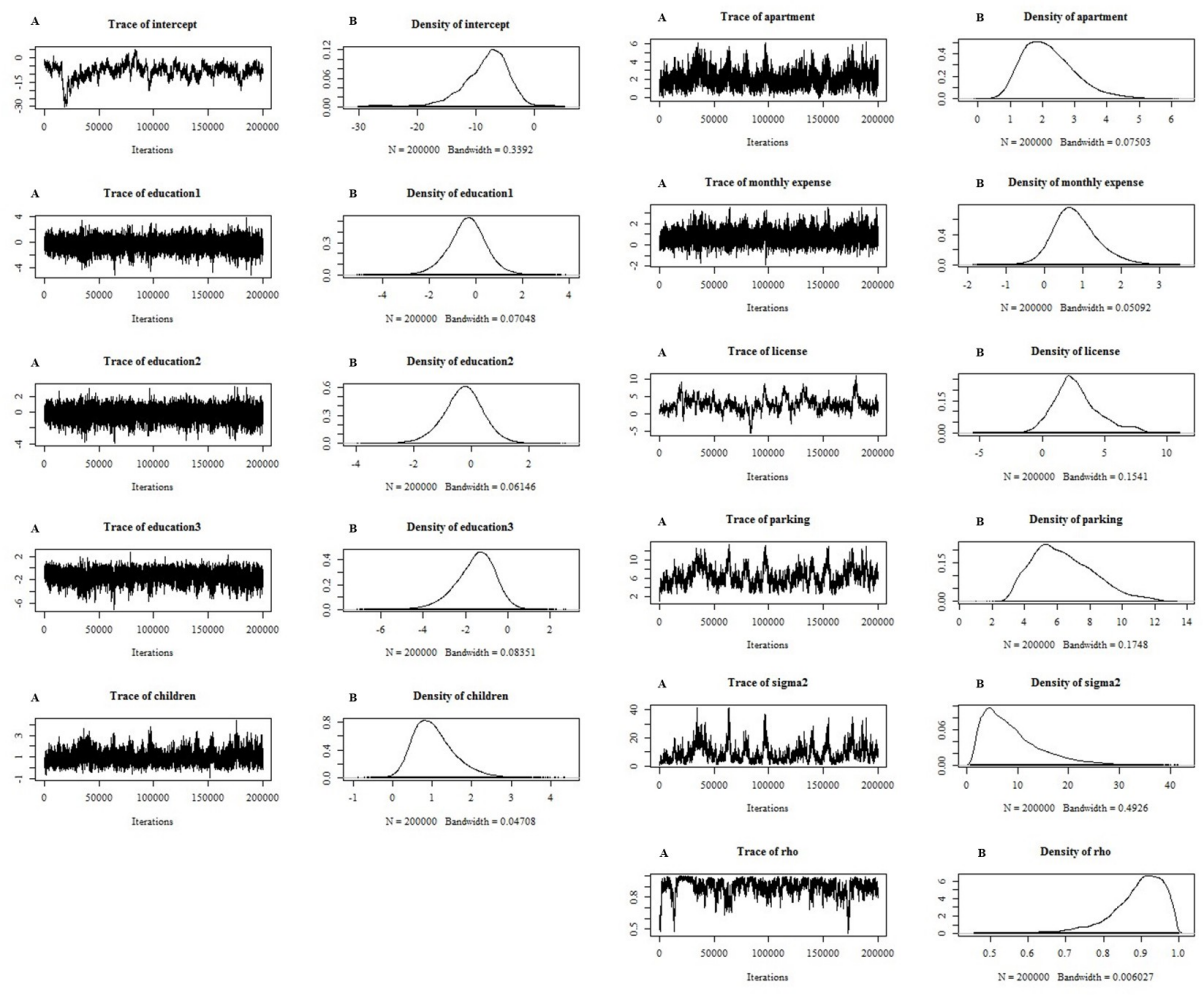
MCMC plots for variables in the spatial autoregressive binary probit model. (A) Iteration of estimated variable value. (B) Probability density distribution of the variable.

In the Bayesian procedure, 200,000 iterations of Gibbs sampling are performed. The first 150,000 sample draws are considered burn-in, and the remaining draws are used to calculate the posterior mean and standard deviation of the draws, which is the standard error of the estimate. Three dummy variables related to education level are introduced into the model. In total, we estimate three models. The first model we test is the pure probit model estimated by maximum likelihood estimation (MLE), and the second is the pure probit model estimated by the Bayesian MCMC method. The third model is a spatial autoregressive binary probit model that considers the interdependence of household private car ownership, and it is estimated by the Bayesian MCMC method. MCMC plots for variables in the third model are described in Fig 1. Estimation results are summarized in Table 2. One thing we need to keep in mind is that we cannot directly compare the estimates from the pure probit model with the estimates from the spatial autoregressive probit model due to differences in the magnitude of the covariance matrix. The entire data set (748 observations) is divided into two subsets. We use the first 600 observations to estimate the model, and the 148 remaining observations are used to perform the hold-out sample test.

**Table 2:** Estimation results.

Coefficient	Probit_MLE	Probit_Bayes	Probit_inter_Bayes
(Intercept)	-2.02(0.279)***	-2.055(0.288)***	-8.300(3.162)***
Education1	-0.066(0.251)	-0.067(0.257)	-0.365(0.866)
Education2	-0.054(0.217)	-0.054(0.224)	-0.271(0.744)
Education3	-0.448(0.262)*	-0.448(0.270)*	-1.600(0.963)*
Children	0.314(0.132)**	0.319(0.134)**	1.113(0.552)**
Apartment	0.788(0.171)***	0.801(0.168)***	2.276(0.829)***
Monthly expense	0.169(0.165)	0.176(0.166)	0.888(0.607)
License	0.605(0.096)***	0.613(0.097)***	2.941(1.873)
Parking	2.081(0.195)***	2.116(0.197)***	6.677(1.903)***
*σ*^2^	NA	NA	9.615(5.534)*
*ρ*	NA	NA	0.867(0.076)***
loglik.	-197.772	-202.3	-63.38
In-sample fit:			
logMargDenNR		-208.023	-145.086
Out-of-sample fit:			
Actual share		62.16%	62.16%
Model share		39.65%	50.35%

Notes

*** p<0.01

** p<0.05

* p<0.1

elements in parenthesis are standard errors.

We employ two indicators, loglikelihood value and log marginal density, to measure in-sample fit. The log marginal density (for details, see [49]) can be calculated with the importance sampling method of Newton and Raftery, and it can be expressed as Eq (12).

$$\hat{p}(y|M_{i})=(\frac{1}{R}\sum_{r=1}^{R}\frac{1}{l(\theta^{r}|M_{i})}{)}^{-1}$$ (12)

Where *y* is the actual data, *M*_*i*_ is the *ith* model, *R* is the total number of posterior draws, *θ*^*r*^ is the *rth* draw of *θ*, and $l(\theta^{r}|M_{i})$ is the likelihood value at the *rth* draw of *θ* conditional on model *M*_*i*_. Out-of-sample fit is measured by the prediction error of owning a vehicle. The approach we employ is the sample enumeration method [50]. Indicators of both the in-sample and out-of-sample are also listed in the bottom of Table 2. The difficulty lies in obtaining values of augmented parameter *θ*_*out*_ for hold-out observations. It was proven that [46]:

$$\left(\begin{array}{l} \theta_{in}\\ \theta_{out} \end{array}\right)\approx N(0,{\hat{\sigma }}^{2}(I_{748}-\hat{\rho }\hat{W}{)}^{-1}(I_{748}-\hat{\rho }{\hat{W}}^{'}{)}^{-1})=N(0,\begin{array}{l} \Sigma_{in-in}\;\Sigma_{in-out}\\ \Sigma_{out-in}\;\Sigma_{out-out} \end{array})$$ (13)

Where *θ*_*in*_ is the vector of augmented parameters for observations that were used to estimate the model, ${\hat{\sigma }}^{2},\hat{\rho }$ are the estimated parameters, $\hat{W}$ is constructed for the total observations, Σ_*in−out*_ is the covariance matrix between *θ*_*in*_ and *θ*_*out*_. We can obtain Σ_*in−in*_, Σ_*in−out*_, Σ_*out−in*_, and Σ_*out−out*_ according to Eq (13). Then, the conditional distribution of *θ*_*out*_ given *θ*_*in*_ can be derived using properties of the multivariate normal distribution [46]:

$$(\theta^{p}|\theta )∼N(\mu ,\Omega ),$$ (14)

Where $\mu =\Sigma_{out-in}\Sigma_{in-in}^{-1}\theta_{in}$ and $\Omega =\Sigma_{out-out}-\Sigma_{out-in}\Sigma_{in-in}^{-1}\Sigma_{in-out}$. Elements of vector *μ* are the values of *θ*_*out*_. Then, we can simulate the choice probabilities for the hold-out sample using the Geweke-Hajivassiliou-Keane (GHK) simulator [51,52].

As shown in Table 2, the model results of pure probit estimated by the MLE and the Bayesian MCMC are remarkably close in magnitude and in significance level. The loglikelihood value indicates that the MLE is slightly better than the Bayesian MCMC method. However, the small difference could be negligible in terms of real applications, which reveals that the Bayesian MCMC estimation is an alternative to the classical estimation for discrete choice models. Hence, we will mainly focus on the comparison between pure probit and spatial autoregressive probit, both of which are estimated by the Bayesian MCMC method.

With respect to the magnitude of the parameter estimates, the spatial autoregressive probit model exhibits some differences due to the reasons discussed previously. However, the significance level is consistent with the pure probit model in regards to the number of driver licenses, which is the indicator for interdependence. This indicates that the endogeneity problem exists in the pure probit model, which is also proven by the statistically significant parameters *σ*^2^ and *ρ*. Both parameters are statistically different from 0, indicating the existence of the preference interdependence. *ρ* is 0.867, suggesting conformity among households in the sample. Based on the spatial autoregressive probit model, knowing the choice of other households is helpful in predicting the choice preference. Comparing the performance of both models, the spatial autoregressive probit model greatly outperforms the pure probit model in both the in-sample fit and out-of-sample fit. Considering the interdependence, the loglikelihood value increases from approximately -202 to approximately -63. The log marginal density also shows a significant improvement, increasing from -208 to -145. The prediction error in the hold-out sample indicated by the spatial autoregressive probit decreased to 18.9% from 36.2%, indicating the effectiveness of the model when considering interdependence, at least compared with the pure probit model under this same scenario. Next, we will focus on analyzing the results from the spatial autoregressive probit model.

The estimates indicate that education plays a nonsignificant role in other subgroups except for graduate level. A statistically significant coefficient shows that people with a graduate education level are less likely to own a vehicle. However, a cautious analysis should be implemented before drawing any conclusions. We cannot directly conclude that people with higher education levels tend to be environmentally friendly since we cannot be sure about their job or career. They may be graduate students or new to their job or career and may be temporarily constrained by income. After they stabilize in their career, they probably would like to own a vehicle. Since we do not have this type of information, we cannot incorporate it into the model. Moreover, the number of children in a family significantly stimulates a household to purchase a vehicle. Children are always at the center of Chinese families, and most activities revolve around the children. Therefore, the need for travel flexibility and comfort in a household with children enhances vehicle owning behavior. The significant impact of the number of driver licenses is captured by the interdependence in the model, resulting in a decrease in the significance level of this variable. Furthermore, owning an apartment or a parking lot is positively associated with private car ownership. This may reveal that Chinese people tend to purchase an apartment first and then buy a vehicle. Monthly expense is not significant for this data set.

An analysis of the differences between the variables for two groups (*θ*>0 and *θ*<0) is summarized in Table 3. Accordingly, we find that parking is statistically different between groups other than the license group, indicating that the augmented parameter that captures the endogenous nature of preference is also associated with parking status.

**Table 3:** Analysis of the augmented parameter.

	*θ*>0	*θ*<0	t_value
Education	1.781 (0.052)	1.733	0.926
Children	0.551 (0.032)	0.504	1.490
Apartment	0.704 (0.025)	0.699	0.200
Monthly expense	0.904 (0.028)	0.929	-0.910
License	1.506 (0.027)	1.451	2.008**
Parking	0.518 (0.027)	0.267	9.167***

## Conclusion

People are connected by various social networks and driven by shared norms and values or similar backgrounds with respect to education, geographical origin, living habits, etc. Therefore, the private car ownership choice of a household may be influenced by neighbors, friends, respected people, or social media in various ways. Ignoring these interdependent relationships may result in inaccurate point estimates and therefore lead to policy bias. Therefore, this paper employs a spatial autoregressive binary probit model, estimated by the Bayesian MCMC method, to investigate the existence of interdependence in household behavior related to private car ownership.

We first estimate and compare two pure probit models using the MLE and Bayesian MCMC methods. The results suggest that point estimates, standard errors, and significance levels are remarkably close, indicating that Bayesian MCMC estimation is an effective alternative to classic estimation methods. However, these estimates may not be very powerful in revealing the actual mechanism of household private car ownership choice since choice interdependence is proven to exist, as shown by the estimation results of the spatial autoregressive probit model. The interdependence parameter *ρ* is significantly positive, indicating a positive correlation among households. Moreover, model fit is greatly improved in both the in-sample fit and out-of-sample fit when the endogenous preference dependence is taken into account. The results revealed by the spatial autoregressive probit model suggest that the number of children and the ownership of an apartment or a parking lot are positively and significantly associated with private car ownership. Moreover, education may play a significant role in reducing private car ownership. A significant influence of the number of driver licenses is captured by the augmented parameter *θ*. Further analyzing the difference between the variables for subgroup *θ*>0 and *θ*<0, we discover that the parking variable is statistically different, suggesting that *θ* is associated with it in addition to the license variable.

The model can be expanded in many ways based on the information and data it contains. First, it can be expanded from binary choice to multinomial choice. Second, in our study, we construct the weight matrix only demographically, but it can also be constructed geographically and demographically, which is called the mixing weight. In this case, a mixing probability parameter can be estimated as well. Third, this weight matrix could be asymmetric, and the asymmetric matrix could be used to imitate the influence degree. Moreover, the weight matrix can be extended to multiple dimensions, including the temporal dimension. Finally, it can be expanded to investigate the interdependence of the point estimates of the variables in the population. These developments will be meaningful in explaining consumer choice heterogeneity, and we will focus on them next.

## Appendix

Conditional Gibbs sampler

1. Given the choice, the latent continuous variable *z* is truncated normally and distributed as
  $$f(z|\beta ,\theta ,y)=\mathrm{truncated}\;\mathrm{normal}(x_{n}^{'}\beta +\theta_{n},1).$$
  Generate {*z*_*n*_,*n* = 1,…,*N*}: If *y*_*n*_ = 1, then *z*_*n*_≥0. If *y*_*n*_ = 0, then *z*_*n*_<0.
2. Generate *β*:
  $$\begin{array}{l} f(\beta |\theta ,z)=MN(\nu ,\Omega )\\ \nu =\Omega (X^{'}(z-\theta )+D^{-1}\beta_{0})\\ \Omega =(D^{-1}+X^{'}X{)}^{-1}\\ \beta_{0}=(0,0,\ldots ,0{)}^{'}\\ D=400I_{N} \end{array}$$
3. Generate *θ*:
  $$\begin{array}{l} f(\theta |\rho ,\sigma^{2},z,\beta )=MN(\nu ,\Omega )\\ \nu =\Omega (z-X\beta )\\ \Omega =(D^{-1}+\sigma^{-2}B^{'}B{)}^{-1}\\ B=I-\rho W \end{array}$$
4. Generate *σ*^2^:
  $$\begin{array}{l} f(\sigma^{2}|\rho ,\theta )\propto Inverted\;Gamma(a,b)\\ a=s_{0}+N/2,(s_{0}=5)\\ b=\frac{2}{\theta^{'}B^{'}B\theta +2/q_{0}},(q_{0}=0.1) \end{array}$$
5. Generate *ρ*:
  We need to use the Metropolis-Hasting algorithm with a random walk chain to generate sample draws. Let *ρ*^(*p*)^ denote the previous draw; then, the next draw *ρ*^(*n*)^ is given by:
  $$\rho^{\left(n\right)}=\rho^{\left(p\right)}+\Delta$$
  With the accepting probability *α* given by:
  $$\min\;\left[\frac{\left|B(\rho^{\left(n\right)})|\exp\{-0.5(1/\sigma^{2})\theta^{'}B(\rho^{\left(n\right)}{)}^{'}B(\rho^{\left(n\right)})\theta \right\}}{\left|B(\rho^{\left(p\right)})|\exp\{-0.5(1/\sigma^{2})\theta^{'}B(\rho^{\left(p\right)}{)}^{'}B(\rho^{\left(p\right)})\theta \right\}},1\right]$$
  Δ is a draw from the normal density (0, 0.005). If *ρ* lies outside the range of $\left[\frac{1}{\lambda_{\min}},\frac{1}{\lambda_{\max}}\right]$, the draw *ρ*^(*n*)^ is rejected.

## Supporting information

S1 Questionnaire Questionnaire in English. (DOCX)

S2 Questionnaire Questionnaire in Chinese. (DOCX)
