# Supplementary material for: Investigation of household private car ownership considering interdependent consumer preference
Source: PLoS One. 2019 Jul 10;14(7):e0219212. doi: 10.1371/journal.pone.0219212 (PMC6619740; doi:10.1371/journal.pone.0219212)
Supplement: S1 Questionnaire — (DOCX) [file pone.0219212.s001.docx]

**Investigation of household private car ownership and usage behavior**

This questionnaire is part of a National Natural Science Foundation of China Project. It is used to study household private car ownership and usage behavior. The reliability and accuracy of your answers are crucial in the statistical analysis of the research. The survey is conducted anonymously, and information you provide here will only be used for research. Thank you for your cooperation.

***

***

**Part 1 Basic information**

1. Gender: ( )

| A. male | B. female |  |  |
| --- | --- | --- | --- |

1. Age: ( )

| A. 18—30 | B. 31—50 | C. 51—65 | D. above 65 |
| --- | --- | --- | --- |

1. Education: ( )

| A. below high school | B. junior college | C. Bachelor’s degree | D. Master’s degree |
| --- | --- | --- | --- |

1. How many Children do you have? ( )

| A. 0 | B. 1 | C. 2 or above |  |
| --- | --- | --- | --- |

1. Do you rent an apartment? Or own your apartment?( )

| A. rent an apartment | B. own an apartment |  |  |
| --- | --- | --- | --- |

1. Household monthly expense: ( )

| A. less than 5,000 CNY | B. 5,000 CNY-10,000 CNY | |
| --- | --- | --- |
| C. 10,000 CNY-20,000CNY | D. more than 20,000 CNY |  |

1. How many driver licenses in your family? ( )

| A. 0 | B. 1 | C. 2 | D. 3 or above |
| --- | --- | --- | --- |

1. Do you have a driver license?

| A. Yes (What was your age when you obtained the driver license? ) | B. no |
| --- | --- |

1. How many private cars do you have in your family? ( )

| A. 0 | B. 1 | C. 2 or above |  |
| --- | --- | --- | --- |

1. Do you have your own parking lot? ( )

| A. yes | B. no |  |  |
| --- | --- | --- | --- |

1. How many people work full-time in your family? ( )

| A. 0 | B. 1 | C. 2 | D. 3 or above |
| --- | --- | --- | --- |

1. Living situation: ( )

| A. live alone | B. couple | C. couple + children | D. couple + parents |
| --- | --- | --- | --- |
| E. couple+children+parents | F. others |  |  |

1. Living area: ( )

| A. Zhongshan district | B. Xigang district | C. Shahekou district | D. Ganjingzi district |
| --- | --- | --- | --- |
| E. lvshunkou district | F. Jinzhou district | G. others |  |

1. Walking time from the living area to the nearest public transport station: ( )

| A. within 5 minutes | B. 5-10 minutes | |
| --- | --- | --- |
| C. 10-20 minutes | D. more than 20 minutes |  |

**Part 2 Private car ownership and usage behavior**

1. Please fill in the form below about each car you own in your household. Please make sure the information you provide is complete.

|  | 1^st^ private car | 2^nd^ private car | 3^rd^ private car |
| --- | --- | --- | --- |
| Purchasing time |  |  |  |
| Brand |  |  |  |
| Displacement |  |  |  |
| Fuel type |  |  |  |
| Initial cost |  |  |  |
| Annual maintenance fee |  |  |  |
| Vehicle miles travelled (unit: km) |  |  |  |
| Source of purchasing funding | Self-pay □  Sponsored □ | Self-pay □  Sponsored □ | Self-pay □  Sponsored □ |

This is the end of the questionnaire.

Thank you again for your cooperation!

If you have any questions，please contact**

Telephone：***
