# Supplementary material for: Investigation of household private car ownership considering interdependent consumer preference
Source: PLoS One. 2019 Jul 10;14(7):e0219212. doi: 10.1371/journal.pone.0219212 (PMC6619740; doi:10.1371/journal.pone.0219212)
Supplement: S2 Questionnaire — (DOCX) [file pone.0219212.s002.docx]

私人汽车保有与使用行为的实证研究

本调查是国家自然科学基金项目的一部分，您回答的真实性/准确性对我们的统计分析至关重要，请您积极配合，万分感谢。本次调查完全匿名，您所提供的信息都会绝对保密，本调查仅用于科学研究，请您放心作答。

***

***

**基本情况调查**

1. 您的性别：( )

| A.男 | B.女 |  |  |
| --- | --- | --- | --- |

1. 您的年龄：( )

| A. 18岁—30岁 | B. 31岁—50岁 | C. 51岁—65岁 | D. 65岁以上 |
| --- | --- | --- | --- |

1. 您的学历：( )

| A. 高中及以下 | B. 专科 | C. 本科 | D. 研究生 |
| --- | --- | --- | --- |

1. 您有几个小孩：( )

| A. 无 | B. 1 个 | C. 2 个及以上 |  |
| --- | --- | --- | --- |

1. 您目前租房还是有自己的房子：( )

| A. 租房 | B. 拥有自己的住房 |  |  |
| --- | --- | --- | --- |

1. **家庭**月均消费有多少：( )

| A. 5千元以下 | B. 5千-1万元，包括5千元 | |
| --- | --- | --- |
| C. 1万-2万元，包括1万元 | D. 2万及以上 |  |

1. 您家有几人有驾照：( )

| A. 无 | B. 1人 | C. 2人 | D. 3人及以上 |
| --- | --- | --- | --- |

1. 您是否有驾照：

| A. 是 (初次获得驾照的年龄是( )岁) | B. 否 |
| --- | --- |

1. 您家有几辆私人汽车： ( )

| A. 0 辆 | B. 1辆 | C. 2辆及以上 |  |
| --- | --- | --- | --- |

1. 是否拥有自己的停车位：( )

| A. 是 | B. 否 |  |  |
| --- | --- | --- | --- |

1. 您家有几人工作：( )

| A. 0 个 | B. 1个 | C. 2个 | D. 3个及以上 |
| --- | --- | --- | --- |

1. 您的居住情况：( )

| A. 独自居住 | B. 夫妇两人居住 | C. 夫妇+孩子 | D. 夫妇+老人居住 |
| --- | --- | --- | --- |
| E. 三代同堂居住 | F. 其他 |  |  |

1. 您目前住在哪里：( )

| A. 中山区 | B. 西岗区 | C. 沙河口区 | D. 甘井子区 |
| --- | --- | --- | --- |
| E. 旅顺口区 | F. 金州区 | G. 其他 |  |

1. 住房到最近公交车站的步行时间：( )

| A. 5分钟以内，包括5分钟 | B. 5-10分钟，包括10分钟 | |
| --- | --- | --- |
| C. 10-20分钟，包括20分钟 | D. 20分钟及以上 |  |

**私人汽车保有与使用情况调查**

1. 若拥有私人汽车，请您按照表格内容填写表格(**请务必保证信息填写完整**)。

|  | 第一辆车 | 第二辆车 | 第三辆车 |
| --- | --- | --- | --- |
| 购车时间 |  |  |  |
| 车辆品牌 |  |  |  |
| 车辆排量 |  |  |  |
| 燃料类型 |  |  |  |
| 初始上路成本(包括**购车成本**及上路保险等) |  |  |  |
| 年均养护费用 |  |  |  |
| 已使用里程(单位：公里) |  |  |  |
| 购车费用来源  (请在对应的选项后打√) | 自付 □  他人赞助 □ | 自付 □  他人赞助 □ | 自付 □  他人赞助 □ |

本问卷到此结束，非常感谢您的合作！

如有疑问，请联系**

联系电话：***
